# Unusual Case of Localized Upper Limbs and Truncal Edema Associated with Fever, Anemia, and Acute Kidney Injury

**DOI:** 10.1155/2019/6251426

**Published:** 2019-11-22

**Authors:** Mohammad Tinawi

**Affiliations:** Nephrology Specialists, P.C., 801 MacArthur Blvd., Ste. 400A, Munster, IN 46321, USA

## Abstract

The patient is a 75-year-old man who presented with right arm pain, edema, and erythema. The same manifestations appeared in the other arm 3 weeks later. He also developed fever, acute kidney injury, anemia, and truncal edema. Initial extensive evaluation was unrevealing. He was noted to have elevated creatine kinase, and a diagnostic muscle biopsy lead to diagnosis of inflammatory myositis. He improved with corticosteroids.

## 1. Introduction

The following is a report and literature review regarding an elusive case of edematous inflammatory myositis. The patient required extensive workup and a lengthy hospital stay.

## 2. Case Presentation

The patient is a 75-year-old Caucasian male who presented with right arm pain, redness, and swelling that started 10 days prior to his admission.

His past medical history is remarkable for hearing loss and age-related macular degeneration. He is a heavy smoker, and his only medication was a daily multivitamin.

His physical exam was remarkable for erythema and 3 + pitting edema of his right upper extremity. The skin of the right upper extremity had a dark maroon ecchymosis-like discoloration without necrosis. Muscle exam of the upper extremities revealed 2/5 strength with the proximal groups. Lower extremities revealed 4/5 strength.

Initial labs showed unremarkable CBC, electrolytes, creatinine, glucose, bilirubin, and alkaline phosphatase. Serum ALT 1.19 *μ*kat/L and AST 3.5 *μ*kat/L was found. Creatinine kinase (CK) was elevated at 53.7 *μ*kat/L (3158 IU/L) on day 1 (normal range 0.34–3.42 *μ*kat/L), and on day 5, it was 32 (1878 IU/L) and remained elevated until it returned to the normal range on day 35.

CT scan of the right arm with intravenous contrast showed nonspecific soft tissue swelling involving the right upper extremity with no abscess.

### 2.1. Hospital Course

The patient was admitted with a diagnosis of right upper extremity cellulitis and treated with antibiotics with no improvement. He had no evidence of upper deep venous thrombosis (DVT).

On day 12, he underwent I&D of the right arm. Serous-like fluid was obtained, and a wound VAC (vacuum-assisted closure) was placed. Fluid cultures were negative. Remarkably, the patient had significant drainage from the incision, 150–600 ml per day. On hospital day 22, the patient had a fever of 39°C and he was noted to have pitting edema and erythema in both upper extremities and truncal edema. Red papules were noted in both arms but no pustules. There was no significant edema in the lower extremities.

On hospital day 25, he had a brief episode of acute kidney injury that resolved with intravenous fluids. He received 2 units of packed red blood cells on day 26 for hemoglobin of 76 g/L.

Serological evaluation including C3, C4, RPR, HIV, hepatitis B and C serologies, ANA, and ANCA was unremarkable. Anti-JO-1 antibody was negative.

On hospital day 30, he underwent a diagnostic muscle biopsy of the left shoulder.

**Figure 1:**
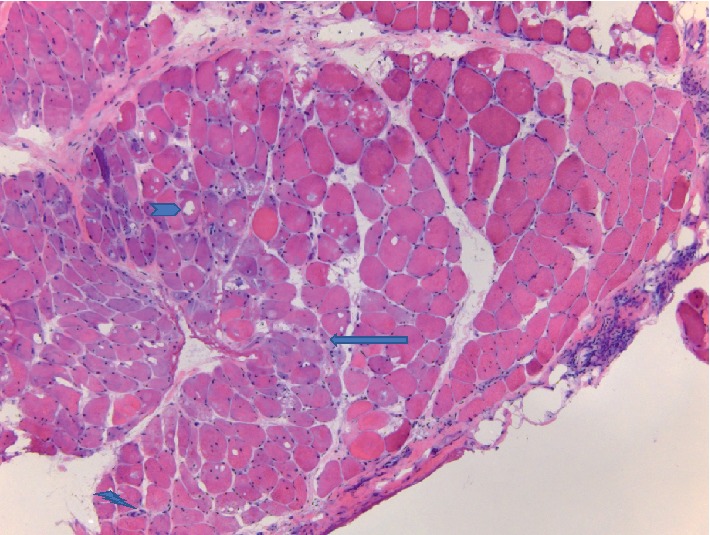
The right side shows more normal muscle. Towards the left half, the muscle contains a number of fibers that have bluer (basophilic) cytoplasm (arrow) and some internal degenerative vacuoles (arrowhead). The left lower corner shows some of the inflammatory cells in the sample (lightning bolt). H&E stain.

**Figure 2:**
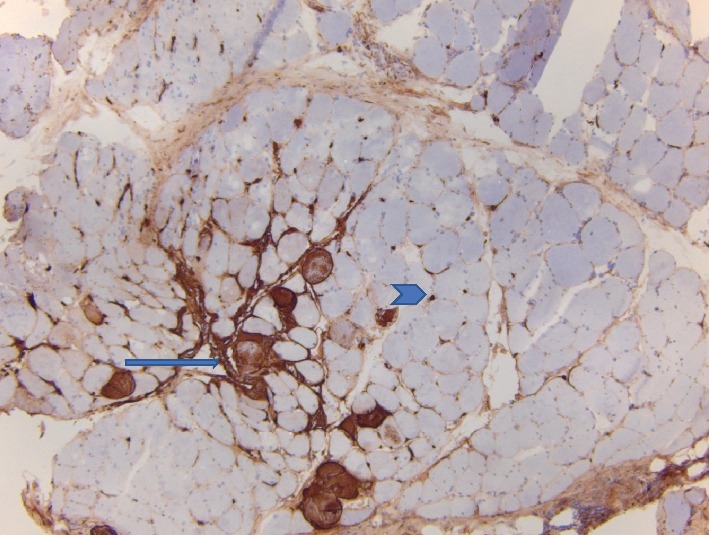
There are clustered fibers with cytoplasmic staining (arrow). Cytoplasmic activation of the membrane attack complex (MAC) is seen in necrotic myofibers due to ongoing necrosis. The background also shows small brown dots between muscle cells (arrowhead). These correspond to capillaries with abnormal expression of the MAC due to capillary damage in dermatomyositis. MAC immunohistochemistry stain.

The biopsy revealed basic features of an inflammatory myopathy. It was consistent with focal perifascicular accentuation of muscle damage and primarily perifascicular distribution of the inflammation. These features are consistent with a diagnosis of dermatomyositis (DM), see Figures 1 and 2. Congo red staining was negative for amyloid. The muscle showed mild changes in fiber-type distribution that could suggest chronic reinnervation changes.

The patient fulfilled several diagnostic criteria for DM including symmetrical muscle weakness in the shoulders and upper arms, elevated CK, fever as a sign of systemic inflammation, and perifascicular atrophy on muscle biopsy. The patient was treated with a tapered course of corticosteroids. On day 53, he was discharged on prednisone 30 mg p.o. b.i.d. After 3 days of treatment with corticosteroids, the patient's edema and rash have resolved. He did not have pulmonary manifestations or evidence of malignancy.

## 3. Discussion

Dermatomyositis (DM) is an idiopathic inflammatory myopathy that involves the proximal muscles and the skin [1]. Inflammatory myopathies (IM) also include polymyositis (PM) and inclusion body myositis. DM is a complement-mediated microangiopathy where antibodies activate the complement system MAC, leading to vascular endothelial damage with subsequent ischemic and micronecrosis of muscle fibers (perifascicular atrophy) [1].

Edema in DM may be due to capillary inflammation and increased vascular permeability [2]. Edema is not considered one of the diagnostic criteria for DM.

This is a case of edematous dermatomyositis affecting one arm and followed by edema of the other arm and the trunk three weeks later. The diagnosis was delayed due to the rarity of DM and not considering muscle weakness and CK elevation at presentation.

Serological evaluation was unrevealing including anti-JO-1 antibody.

DM is a rare condition, and subcutaneous edema especially without typical skin manifestation is a rare presentation of this IM. In hindsight, a diagnostic muscle biopsy should have been obtained at the time the seroma in the right arm was drained.

Symptoms and CK quickly improved with corticosteroids. The appearance of DM on the muscle biopsy is characteristic, and it helps in its differentiation from another IM such as PM.

The fever and anemia were due to the autoimmune disease. The absence of anti-JO-1 antibody does not rule out the diagnosis of DM.

The patient did not have the classic dermatological manifestations of DM such as heliotrope rash, periungual telangiectasias, or Gottron's papules.

The persistence of drainage from the wound VAC placed on the right arm is another peculiar manifestation resulting from severe edema of subcutaneous and muscular tissues.

Milisenda et al. [3] reviewed 19 cases of edema associated with DM. That was the total number of cases reported up to 2014. Of the 19 patients, only 3 had no other skin manifestations of DM.

The review concluded that edematous DM is a rare form of the disease, affecting less than 6% of the patients. Edema may be a sign of an aggressive process that requires prompt treatment.

Most patients required corticosteroids, intravenous immunoglobulin (IVIG), and other immunosuppressive agents.

Werner de Castro et al. [4] reported a case of a 40-year-old man with DM and typical skin manifestations. After 3 months of treatment, he developed anasarca and his skin lesions worsened. Despite aggressive treatment, the patient died due to pneumonia.

Gorelik et al. [5] reported two cases of dermatomyositis with severe subcutaneous edema mimicking deep venous thrombosis. The first patient had severe nonpitting edema in the left forearm. The edema quickly involved the trunk and all limbs. He eventually responded to IVIG and corticosteroids. The second patient had severe pain and edema limited to the left forearm and recovered with supportive treatment.

Tu el al. [6] reported a case of a 38-year-old man who presented with upper body edema and later developed the typical features of DM. The authors reviewed 23 cases of edema associated with DM and PM. Eight patients had edema in all limbs, 3 patients had generalized edema, 2 patients had edema localized to the forearms, and the other 10 had edema localized to the upper limbs with or without facial and truncal edema. Most patients received immunosuppressive therapy. Three patients died. The authors concluded that subcutaneous edema can be the first presenting sign of DM and is associated with significant morbidity and mortality.

## 4. Conclusion

Subcutaneous edema is a rare manifestation of DM. It can be the only skin manifestation and is currently not part of the diagnostic criteria for DM. Due to the rarity of this presentation, the diagnosis and the treatment are often delayed. The presentation may mimic cellulitis or deep venous thrombosis. Early muscle biopsy and aggressive treatment with immunosuppressive medications are critical.

Edema due to DM is not part of the usual differential diagnosis of edema. It should be considered in patients with isolated limbs or facial edema.
